# Knowing Where to Draw the Line: Perceptual Differences between Risk-takers and Non-Risk-Takers

**DOI:** 10.1371/journal.pone.0091880

**Published:** 2014-03-17

**Authors:** Adam T. Biggs, Paul C. Stey, Christopher C. Davoli, Daniel Lapsley, James R. Brockmole

**Affiliations:** 1 Duke University, Durham, North Carolina, United States of America; 2 University of Notre Dame, Notre Dame, Indiana, United States of America; 3 Central Michigan University, Mt Pleasant, Michigan, United States of America; Anhui Medical University, China

## Abstract

There are a variety of reasons someone might engage in risky behaviors, such as perceived invulnerability to harm or a belief that negative outcomes are more likely for others than for oneself. However, these risk-taking biases are often measured at a decision-making level or from the developmental perspective. Here we assessed whether or not risk-taking influenced perceptual judgments associated with risk. Participants were provided an objective task to measure individual differences in the perception of physical dimensions (i.e., actual size of a balloon) versus the perception of risk (i.e., size at which the balloon would explode). Our results show that specific differences in risk-taking personalities produce specific differences in perceptual judgments about risk, but do not affect perception of the actual dimensions. Thus, risk-takers differ from non-risk-takers in the perceptual estimations they make about risks, and therefore may be more likely to engage in dangerous or uncertain behaviors because they perceive risks differently.

## Introduction

Risk appraisal is a critical factor when someone is trying to decide whether or not to engage in a potentially risky behavior. The specific behavior could range from drug use, to sexual activity, to dangerous driving, but the common component is that the individual must weigh the risk and deem it acceptable or not according to his or her own personal criteria. Although there are numerous influences that might encourage someone to engage in risky behaviors, three of note are *subjective invulnerability,* an *optimism bias*, and *sensation seeking*. Subjective invulnerability has been interpreted as a narcissistic response to developmental challenges [1]–[4], where an individual engages in risky behavior due to a felt sense of invulnerability to injury, harm, and danger. For example, people might drive aggressively because of a belief that they would safely walk away from any accident. Optimism bias represents an individual's belief that he or she is at lesser risk of experiencing a negative outcome as compared to other people [5]–[7]. For example, people might drive aggressively because they believe an accident is more likely to happen to other people than to themselves. Subjective invulnerability and optimism bias are somewhat related constructs with controversial theoretical boundaries [8], [9] that we do not intend to resolve here. Rather, we utilize both constructs in order to more fully capture the range of individual differences that might influence risk perception. The third construct of interest is sensation-seeking [10], and there is a robust literature that links individual differences in sensation-seeking to risk behavior [11].

Hence, subjective invulnerability, optimism bias, and sensation seeking are constructs that describe a disposition to engage in risk-taking behaviors. In this study, we ask whether these biases are related to perceptual judgments—how an individual perceives and then judges physical aspects of the real world such as length or motion—that might underlie risk taking. In the case of aggressive driving, how one appraises risk may depend upon one's perception of the physical world. For example, one might execute an aggressive lane change if one underestimates how fast another car is moving. Some evidence already suggests that reduced sensitivity to certain visual effects can exacerbate risk-taking behaviors; in one study, children who had difficulty determining the speed of an approaching car were more willing to run across the street [12]. An alternative interpretation is that the hypothesized effects occur at the post-perceptual level; that is, risk takers make faulty *predictions* about what they perceive, in spite of ordinary functioning of perceptual mechanisms. For example, riskier driving may stem from an underestimation of the time-to-contact between cars given their relative speeds. The key difference between these two possibilities is whether the decision to engage in risky behavior is due to perceptual estimations about the physical world or perceptual estimations about the possible risk. Thus, our primary aim is to determine whether individual differences in risk-taking biases influence perceptual estimations about physical objects, perceptual estimations about the point of risk, or both.

To examine this question, we used an objective risk-taking scenario in which observers provided an estimation of the actual size for various balloons (to assess perception for physical size), and an estimation of how much larger the balloon could become before popping (to assess what the individual deems to be the point of risk for each balloon). These different measures allowed us to dissociate between what each observer actually saw and what each observer defined as risky. We compared how estimations of risk changed as circumstances neared the point of risk (i.e., popping)—given balloons of different physical size but the same possible volume, the point of risk is father away when the balloon is smaller in actual size and closer when the balloon is larger in actual size. To establish differences in risk-taking personality traits among our participants, we provided three self-report measures of risk-taking: *optimism bias* represents beliefs about the likelihood of danger befalling an individual, *subjective invulnerability* represents beliefs about one's susceptibility to danger, and *sensation seeking* represents the likelihood of an individual putting themselves in a risky situation. We also provided an objective risk-taking measure (i.e., based on task performance and not self-report) by administering the Balloon Analog Risk Task (BART) [13].

Our primary research question is whether any of these risk-taking indices relate to perceptual estimations (i.e., estimates of size, length, etc.). In particular, do risk-takers perceive the physical properties or dimensions of the world differently than non-risk-takers (e.g., “How fast is that car moving?”), or do risk takers differ in their calculation of risk than non-risk-takers (e.g., “Can I get across the street before the passing car?”). The critical distinction is between appraisal of physical properties and appraisal of risk. If there are differences between risk-takers and non-risk-takers for perceptions of the physical world, then these differences would be evident in estimations about the physical size of objects. If there are differences between risk-takers and non-risk-takers in risk appraisal, then these differences would be evident in estimations about the point of risk. Because the provided tasks involved balloons, physical size was represented via the actual size of a real balloon and the point of risk was represented as the size at which the balloon would explode.

## Method

### Ethics Statement

All participants provided written consent to participate in the experiment, and permission to conduct the experiment was obtained from the University of Notre Dame's Human Subjects Institutional Review Board.

### Participants

Sixty-four undergraduate students from the University of Notre Dame participated for partial completion of a course requirement. Data from two participants were removed due to a failure to follow instructions.

### Survey Measures

Three separate self-report measures of risk-taking were collected: optimism bias, subjective invulnerability, and sensation seeking. For the optimism bias, participants rated their chances of experiencing 22 conditional risks compared with the average University of Notre Dame student [8]. Response options ranged between “Much below average (-3)” and “Much above average (3)” on a seven-step scale. Following the precedent of previous research [14]–[16], we included both optimism bias subscales. One subscale included the sum of negative items (19 items; e.g., “Losing a friend because of something I did”), whereas the second subscale included the sum of positive risk items (3 items; e.g., “Getting an interview if I apply for a job”). Note that optimism bias for negative items is normally demonstrated by negative scores (i.e., an individual believes that he or she has below average risk of experiencing a negative outcome), whereas optimism bias for positive risks is demonstrated by positive scores (i.e., an individual believes that he or she has above average chance of a positive outcome). However, the current statistics were recoded such that a larger number indicates a greater optimism bias.

We assessed subjective invulnerability with the Adolescent Invulnerability Scale (AIS) [17]. The AIS assesses self-reported invulnerability with respect to two factors: danger invulnerability (12 items) and psychological invulnerability (8 items). Danger invulnerability represents a perceived resistance to physical danger, whereas psychological invulnerability represents a perceived resistance to personal or psychological distress. Items are rated on a five-step scale ranging from 1 (*Strongly Disagree*) to 5 (*Strongly Agree*).

We assessed sensation seeking by using the Brief Sensation Seeking Scale [18], [19]. Participants completed 8 items by rating each from 1 (*Strongly Disagree*) to 5 (*Strongly Agree*), which could be divided into four different categories (experience seeking, boredom susceptibility, thrill and adventure seeking, or disinhibition).

Aggregate scores were summed for each of these scales (optimism bias, subjective invulnerability, and sensation seeking) and used as a participant's individual score for each attribute. Larger scores indicate that an individual exhibits more of these particular qualities (e.g., larger scores on the sensation seeking scale indicate that the individual is more likely to seek out sensational activities). All survey measures demonstrated adequate reliability in the current sample (optimism bias: α = 0.69; subjective invulnerability: α = 0.83; sensation seeking: α = 0.76), and required approximately ten minutes to complete.

### Balloon Analog Risk Task

Survey measures provided a subjective estimate of an individual's risk-taking tendencies—specifically, the personality traits associated with risk-taking; however, but to provide an objective measure of risk-taking behavior, we included a well-replicated task known as the Balloon Analog Risk Task (BART) [13]. Participants completed this task on a computer, which was based on balloon inflations. Participants received $0.05 per successful pump to inflate the balloon, and on each trial, participants had the option of either risking another pump to gather more money or to collect the money already accumulated. The money accumulated per balloon was lost if the balloon exploded. Data was collected on the BART task for 54 of the 62 participants in this study (eight were lost due to computer error), and each participant took approximately fifteen minutes to complete the BART.

There were 29 different balloons which each exploded at a random point, and the dependent variables of interest were the number of pumps per un-popped balloon and the number of popped balloons. The BART task typically includes 30 different balloons, although computer error caused the last trial of each session to be lost—thus leaving 29 total balloons. Number of pumps per balloon was calculated as an adjusted average, incorporating only the average number of pumps per balloon for balloons that did not explode. Number of popped balloons indicated how many of the 29 balloons exploded per participant during the experiment. Because the balloon explosions are based upon a random principle rather than physical constraints, the BART is used as an objective measure of risk-taking. Risk-takers are more likely to continue pumping each balloon without collecting the reward, which generates a larger average number of pumps per balloon and more balloon explosions.

### Balloon Estimations Task

**Figure 1:**
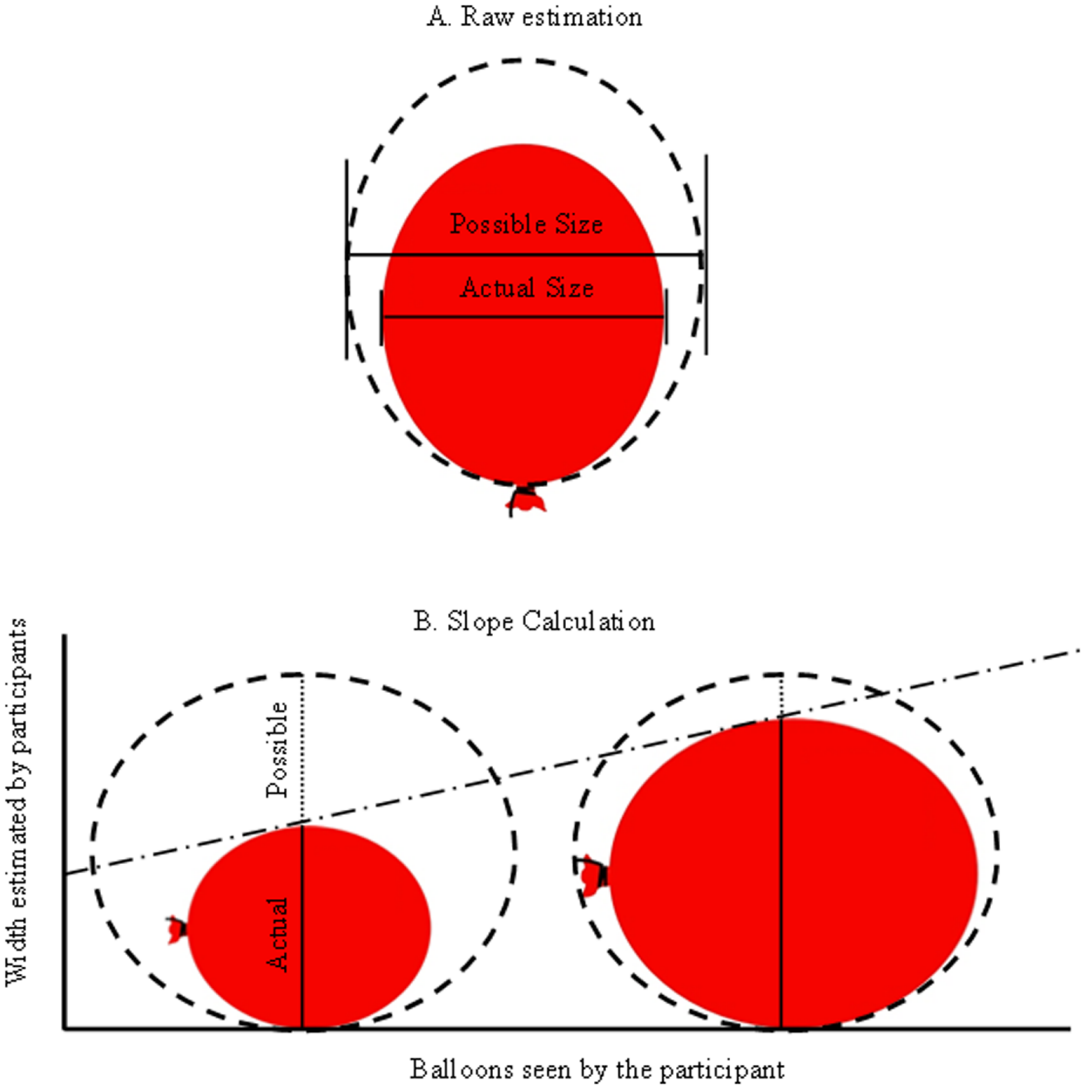
Graphical depictions of the calculations. (**A**) Participants made estimations about the actual size of each particular balloon and how big it could become before popping. These estimations provide a measure of both real-world perception and the subjective point of risk (i.e., popping for balloons) for each participant. (**B**) We calculated the slope changes based upon the estimations reported by each participant. The actual balloons varied in size (either 4, 5, 6, 7, or 8 inches wide), and we plotted these actual sizes against the estimated sizes reported by the participant. Note that actual sizes should show a linear increase of 1 if the participant reported actual sizes with perfect accuracy, whereas additional size estimates should show a linear decrease of -1 if the participant reported additional sizes with perfect accuracy.

To compare the potential perceptual differences between risk-takers and non-risk-takers, we created a task with an objective point of risk that could be completed in a laboratory setting. In the balloon estimations task, participants made judgments about the physical size of real balloons—not the computer-based balloons of the BART. Each participant was seated across a table from the experimenter, and provided estimates about the physical dimensions of different balloons. The first estimation concerned the actual width of the balloon at its widest point, and the second estimation concerned how much additional width could be added before the balloon exploded (see Figure 1). The actual width estimation provided an assessment of perceptual judgments for physical qualities in the real world, whereas the additional width assessment provided an assessment of perceptual judgments for an objective point of risk. All participants except one reported these dimensions in inches, and metric responses reported by one participant were converted to inches.

Participants also reported length estimates of the balloon; however, our analyses focus solely upon the maximum width of the balloon in order to avoid any confusion as to whether the length of the balloon included the tied-off portion. For example, participants may have altered their estimates of balloon length to include the tied-off portion, participants could have been influenced by the experimenter's hand obscuring part of the balloon, or there could have been derivations between balloons as to the precise size of the tied-off portion. Because these possibilities could influence the results in unpredictable and uncontrollable ways, we focused only on the width estimations as balloon width was clearly in view without an asymmetrical portion (i.e., some tied-off length).

Experimenters held the balloon by its tied-off portion with an arm extended flat and resting on the table. Only one balloon was shown at a time, and returned to a spot obscured from the participant's viewpoint before the next balloon was shown. Participants were never allowed to handle the balloons. Each balloon had one of two possible sizes (e.g., the balloon had a maximum of 10 or 14 inches in width before popping), and five different actual sizes were created for each possible volume (4, 5, 6, 7, and 8 inches wide). Different actual sizes were used to gather an accurate estimation of the perceptual judgments about each balloon; by comparison, if all balloons were the same actual size, participants could have completed the experiment without varying their answers. Additionally, different actual sizes allowed us to analyze how participants' responses changed based upon how close the actual balloon was to popping. For example, given the same possible volume, small balloons are much further from the size at which they would pop (i.e., the objective point of risk) than larger balloons which are closer to the size at which they would pop. In total, participants made judgments about ten different balloons, which also varied in color (white, red, pink, powder blue, and dark blue). Participants always completed the balloon estimations task after the BART task to avoid any influence of the real balloons upon judgments for the computer-generated balloons.

As an example of the full procedure, the experimenter would retrieve and hold up balloon A (dark blue in color, actual width 6 inches) and ask the participant to estimate the physical width of this balloon. Then, after recording the response, the experimenter would ask the participant to estimate how much larger the balloon could become (i.e., additional size) before popping. The experimenter would then return balloon A to its original location out of the participant's view, and repeat the process with balloon B.

### Planned Analyses

We were primarily interested in two different aspects about the size estimations for the physical balloons. The first aspect involved the raw estimations made about each balloon. This approach provides a comparison between the physical dimensions of an object and the person's perception of those dimensions. The variable was calculated for actual size by comparing the difference between the physical width and the participant's estimation; for example, if the physical balloon was 6-inches in width, then a participant reporting 6 inches would be accurate (6-6; scored as a difference of 0) and a participant reporting 4 inches would be inaccurate (6-4; scored as a difference of -2). The raw estimations also involved the additional size estimations made about each balloon. This approach provides a comparison between the possible size of the balloon (i.e., where the balloon would pop, or the objective point of risk) and the person's perception of that risk. The variable was calculated for additional size by adding the actual width to the participant's reported additional width to control for differences in actual size; for example, if the physical balloon was 6-inches in width, then a participant reporting 8 additional inches possible would be accurate for a balloon of 14-inches possible size (14 – (6+8); scored as difference of 0) and a participant reporting 4 additional inches possible would be inaccurate (14 – (6+4); scored as a difference of -4). Thus, both the actual estimations and the additional size estimations are measured via difference scores (to control for either the actual size or the potential size for a given balloon), and perfect estimations yielded a score of zero. Negative scores for actual size indicated an underestimation for physical size, whereas negative scores for additional size indicated an underestimation for the point of risk.

The second aspect we assessed involved the change in estimations with increasing physical size of the actual balloon. This approach provided an assessment of how risk perception changed along with the physical dimensions and proximity to risk. For our earlier example of aggressive driving, the change in estimations is akin to whether a driver is more or less likely to make an aggressive lane change when another car is further away versus much closer. For balloons, there is an objective point of risk in that the balloon has a set maximum size before it will explode. Participants may be more likely to overestimate this point of risk when it is far away (i.e., the physical balloon is small) versus when the point of risk is much closer (i.e., the physical balloon is very large). Thus, change in estimations across the actual physical sizes provided an assessment as to whether an individual's risk assessment was similar in all cases or dependent upon proximity to the point of danger. Slope calculations were determined for each individual by using the physical size of the actual balloon as the x-axis values, his or her estimations as the y-axis values, and plotting a line of best fit for each individual. Given that the five actual sizes of the balloons each differ at an interval of one inch, perfectly consistent change across the actual sizes of the balloons would have a slope of positive one for the actual estimations and a slope of negative one for the additional estimations. Shallower slopes represent less change across physical dimensions, whereas steeper slopes represent more change across physical dimensions. For example, someone who overestimates the point of risk for small balloons (e.g., a real balloon 4-inches in width could have 12 inches added to its width) might also overcorrect their estimations for larger balloons (e.g., a real balloon 8-inches in width could have 1 inch added to its width), which would likely produce a steep slope function across all balloon sizes.

Our analyses will primarily be done with two statistical methods. First, we will use correlations to determine potential relationships between measures. Second, in the case where multiple factors correlate with a single dependent measure (e.g., accuracy of actual size estimations), we will use regression models to determine how the factors interact, and which among them best predicted variance within the dependent measure.

## Results

Raw estimations of actual size significantly correlated with the actual size slope estimations [*r*(60)  = −0.67, *p*<.001], and, additional size raw estimations significantly correlated with the additional size slope estimations [*r*(60)  = −0.47, *p*<.001]. We expected some correlation between these measures as they are calculated from the same data and dependent upon certain physical limitations (i.e., there is a direct relationship between how much larger the balloon can become and how large it is currently). Despite the robust correlations, a large portion of the variance remains unexplained (55% and 79%, respectively, based upon the adjusted *R^2^* values of the corresponding regression models), which points to some conceptual independence of the two dependent variables.

### Survey Measures

We first examined the relationship between our various survey measures to ensure that these self-report indices of risk-taking behavior replicated previous findings. See Table 1 for descriptive statistics of all measures, and see Table 2 for correlations between scales. There was a significant relationship between danger invulnerability and psychological invulnerability, *r*(60)  = 0.63, *p*<.001, and as expected, these two indices of subjective vulnerability were positively related. Optimism bias was also significantly related to both danger invulnerability, *r*(60)  = 0.26, *p*<.05, and psychological invulnerability, *r*(60)  = 0.29, *p*<.05, such that a greater optimism bias was also indicative of increased subjective invulnerability for each subscale. These findings replicate previous work using these scales [8]. Of these measures, sensation seeking was only related to danger invulnerability, *r*(60)  = 0.27, *p*<.05, with higher reported scores of sensation seeking also indicating higher reported scores of danger invulnerability.

**Table 1:** Descriptive statistics for key measures.

Variable	Mean	Std. Dev.	Minimum	Maximum
BART Average # of pumps	29.51	14.94	2.11	72.82
BART Balloon pops	7.2	3.57	1	15
Danger invulnerability	29.5	7.08	13	47
Psych. Invulnerability	21.21	6.31	10	37
Optimism Bias	13.27	11.99	−13	52
Sensation Seeking	24.95	5.56	14	39
Actual Size Estimation	0.34	1.08	−2.46	3.15
Additional Size Estimation	−2.65	1.34	−4.50	1.90
Actual Slope Calculation	1.13	0.34	0.3	1.85
Additional Slope Calculation	−0.67	0.36	−1.95	−0.1

**Table 2:** Correlations between self-report surveys for subjective invulnerability, optimism bias, and sensation seeking.

	DI	PI	OB	SS
Danger invulnerability	-	0.63**	0.26*	0.27*
Psych. Invulnerability		-	0.29*	0.22∧
Optimism Bias			-	0.05
Sensation Seeking				-

∧ *p*<0.1; * *p*<0.05; ** *p*<0.01.

### Raw Estimations

We next examined the relationship between the various risk-taking measures and raw estimations made about the physical balloons. See Table 3 for the relationships between survey measures and the raw estimations. None of the measures correlated with the actual size estimations (all *p*>0.35). However, there were several significant relationships between the risk-taking measures and the additional size estimations. For the BART task, the average adjusted pumps was related to the additional size estimations made about the physical balloons, *r*(52)  = 0.34, *p*<.01. Participants who popped more balloons on the BART task also reported larger additional size estimations about the physical balloons, *r*(52)  = 0.33, *p*<.05. The BART task and the balloon estimation task are inherently similar, and so some correlation is expected; however, balloons in the BART task pop at random intervals, whereas balloons in our physical estimation task were tied to realistic physical possibilities.

**Table 3:** Correlations between measures of risk-taking and responses from the balloon estimation task.

	Raw Estimation		Slope Calculation	
Risk-taking Measure	Actual	Additional	Actual	Additional
BART Average # of pumps	−0.03	0.34*	0.22	−0.27*
BART Balloon pops	−0.09	0.33*	**0.26∧**	−0.29*
Danger invulnerability	−0.09	0.08	0.01	−0.27*
Psych. Invulnerability	−0.06	0.15	0.12	−0.17
Optimism Bias	0.12	0.25*	0.04	−0.20
Sensation Seeking	−0.10	0.13	0.16	−0.27*

∧ *p*<0.1; * *p*<0.05.

The optimism bias was also related to the additional size estimations, *r*(60)  = 0.25, *p* = .05, which appears to be driven more by the positive bias, *r*(60)  = 0.29, *p* = .02, than the negative bias, *r*(60)  = 0.21, *p* = .10. As participants believed there was a smaller chance that bad things would happen to themselves versus others, the point at which participants believed the balloon would explode also increased. This evidence suggests that the optimism bias extends even into the perceptual judgments an individual makes regarding risky outcomes. No significant relationship was observed between additional size estimations and danger invulnerability, psychological invulnerability, or sensation seeking (all *p*>0.2).

### Slope Calculations

Finally, we examined the relationship between our various risk-taking measures and the changes in perception across various physical sizes via slope calculations. See Table 3 for the relationships between survey measures and the slope calculations. Similar to raw estimation analyses, none of the risk-taking measures were significantly related to actual size slope calculations (all *p*s >0.05). The most closely related measure was the number of balloons popped on the BART task and the actual size slope calculation, although it remained only marginally significant, *r*(52)  = 0.26, *p* = .053. However, the adjusted average pumps on the BART task was negatively correlated with the slope of additional size calculations, *r*(52)  = −0.27, *p*<.04. The number of balloons popped during the BART task was also negatively correlated with the additional size calculations, *r*(52)  = −0.29, *p*<.05. Both results from the BART task suggest that participants who were more likely to engage in risk on the BART showed steeper changes in additional size slope calculations.

Two self-report measures were significantly related to the additional size slope calculations: danger invulnerability *r*(60)  = −0.27, *p* = .03; and sensation seeking, *r*(60)  = −0.27, *p* = .04. The sensation seeking difference was primarily driven by the thrill seeking subscale, *r*(60)  = −0.39, *p*<.01, as all other subscales were not significantly related to the slope change for additional size (*p*>0.1). All four factors (adjusted average pumps on the BART task, number of balloons popped during the BART task, danger invulnerability, and sensation seeking) were negatively correlated with additional size slope calculations, indicating that the slope calculation became steeper as each of these factors increased; or, that the change in estimations across sizes was larger for participants who were more likely to engage in risk-taking.

For these risk-taking measures (BART average adjusted pumps, BART number of balloons popped, danger invulnerability, and sensation seeking), we entered all four factors into a stepwise linear regression model to determine which factor best predicted the change in risk assessment for the balloon estimations task; only danger invulnerability emerged as a significant predictor (Adj. *R* ^2^  = .09, β = −.332, *p* = .01). This evidence suggests that people who report higher subjective invulnerability to danger are more likely to change their estimations across the physical dimensions. Although it could result from underestimating the additional size for the larger physical balloons, it is less likely under the present circumstances given that additional size estimations quickly approach a floor effect at larger actual sizes (i.e., if the physical balloon is close to popping, the additional size possible approaches zero). The more probable explanation is that people reporting higher subjective invulnerability substantially overestimate the possible size when looking at smaller physical balloons, although these estimations move toward more normal estimations as the physical balloon becomes larger—ultimately resulting in more accurate estimations when the point of risk is near. In effect, these people substantially underestimate the point of risk when it is far away and overcorrect as the point of risk approaches. These estimations could make someone prone to risky behaviors if the decision to engage in said behavior occurs when the immediate situation does not impose some risk.

## Discussion

The current study investigated whether risk-taking biases could be evident in perceptual judgments. We used balloon estimations as a safe, laboratory-based risk-assessment task because the point where the balloon popped provided an objective estimate of risk. To that end, we were concerned with two primary estimations about the balloon: how the individual judged the actual versus additional size for each balloon, and how those estimations changed as the physical balloon sizes became larger (i.e., closer to the point of risk). These variables provided assessments of an individual's perceptual abilities for real objects, where an individual believes the point of risk to be, and how those assessments changed based upon proximity to risk.

None of the risk-taking measures (BART, optimism bias, danger invulnerability, or sensation seeking) predicted individual variability in estimates of physical proportion for real balloons. However, optimism bias was significantly related to perceptual judgments about the point of risk. Essentially, as participants believed that bad things were less likely to happen to them, their estimation of how large the balloon could be before popping increased. Thus, the optimism bias can be expressed as a perceptual difference as well as a belief about the likelihood of some negative event occurring. This evidence suggests that someone with a substantial optimism bias might be more willing to engage in risky behaviors because, in part, they perceive the risk differently than a non-risk-taker. Furthermore, danger invulnerability was linked to how an individual's perception of risk changed as the point of risk approaches. An individual may underestimate how dangerous a situation can be when the risk is not relatively imminent, but that perception changes quickly as the point of risk approaches. This finding suggests that individuals with higher subjective invulnerability may be more likely to consent to potentially dangerous activities when the threat is not imminent. Thus, an individual might be more likely to set out on a path leading to risky behaviors depending upon when the decision is posed to them—which, for the present study, is akin to showing participants a small versus a large physical balloon.

Prior evidence has explained risk-taking behaviors through both cognitive and developmental means. For example, individuals may develop feelings of invulnerability as a consequence of their personal choices, and ultimate development throughout adolescence [1]–[4], which views this risk-taking bias as forming through developmental means—essentially, surviving a myriad of risky situations with minimal negative consequences could lead an individual to believe he or she is less vulnerable to harm. These feelings of invulnerability could then lead to risky behavioral decisions, such as greater perceived behavioral control over driving after alcohol use [20]. Another approach describes the general premise of invulnerability through an optimism bias [5]–[7], which suggests that individual biases in decision-making are responsible for subjective invulnerability. The present study provides evidence that perceptual judgments can also be prominent cognitive factors in risk-taking behaviors, and different risk-taking personality factors influence different aspects of perceptual judgments (e.g., the optimism bias influenced perception for the objective point of risk, whereas subjective invulnerability influenced how perception of risk changed as the point of risk neared). Namely, people may not make decisions in spite of evident danger, but because their perceptual judgments do not adequately represent the point of risk. Therefore, to use the example from our introduction, individuals may drive aggressively because they are inappropriately judging an important safety factor, such as the space necessary—or the space available—to merge between two other cars. The critical impact is that inaccurate perceptual judgments can lead to a poor decision to engage in risky behaviors.

Future research will need to dissociate of how perceptual judgments can influence decisions in a wider variety of risky situations, although our current findings contribute to the larger discussion of risk-taking behaviors by demonstrating that perceptual differences can underlie the decision to engage in risky behavior. It is one of the primary limitations of this study that we did not manipulate different kinds of risk, nor engage participants in a risk-related behavior. For example, another approach might have been to have participants inflate the balloon themselves until they are comfortable that the balloon could fill no further. However, our goal was to investigate whether there was a link between personality factors and perceptual judgments, not the full extent of that link. Additional studies are necessary to investigate additional personality factors that might influence perceptual judgments now that such a link has been established.

In conclusion, this study extends the evidence that perceptual judgments are subject to a myriad of influences. For example, people tend to perceive the world around them based upon affordances—or possibilities for action [21]–[26], and action-specific differences have been shown to exist in attentional biases as well in perception [27]. Here we demonstrated how personality differences in risk-taking could produce differences in the perceptual judgment of an objective risk. These differences did not manifest in perceptual estimations for actual sizes, which suggests that the differences are specific to risk-taking scenarios (or any similar mental transformation or prediction of future events) and not general differences in perceptual abilities. As such, someone might be more likely to engage in risky behavior because they do not properly perceive the factors related to the risk. Our findings could help risk-takers—individuals predisposed towards risky behaviors—by helping them understand that their actions and decisions may be based upon inaccurate perceptual judgments, although the theoretical value of this research is in the evidence that differences in personality can also yield differences in perception. Ultimately, perception can play an important role in the decision to engage in potentially risky behaviors.
